# Evaluation of Mechanical Energy Consumption in WPC Production from Pine (*Pinus sylvestris*) and Hemp (*Cannabis sativa* L.) with ABS Thermoplastic Additions

**DOI:** 10.3390/ma17215177

**Published:** 2024-10-24

**Authors:** Kamil Roman, Emilia Grzegorzewska, Katarzyna Fedorowicz, Jakub Michalczewski

**Affiliations:** 1 Department of Technology and Entrepreneurship in the Wood Industry, Institute of Wood Sciences and Furniture, Warsaw University of Life Sciences—SGGW, 159 Nowoursynowska Str., 02-776 Warsaw, Poland; kamil_roman@sggw.edu.pl; 2 Faculty of Wood Technology, Warsaw University of Life Sciences—SGGW, 159 Nowoursynowska Str., 02-776 Warsaw, Poland; 206516@sggw.edu.pl (K.F.); 207518@sggw.edu.pl (J.M.)

**Keywords:** wood-plastic composites, polymer, wood chips, biocomposite, hot water extraction

## Abstract

This study investigates lignocellulosic biocomposites’ physicochemical properties and strength parameters with varying thermoplastic content. Biocomposites were prepared using wood (*Pinus sylvestris*) or hemp shives (*Cannabis sativa* L.) combined with 25% and 50% ABS regranulate. The research focused on evaluating the mechanical energy consumption during the compaction of wood-ABS biocomposites with different pine fractions pretreated with hot water extraction (HWE) and analyzing the relationship between strength and thermoplastic content. Results indicate that the composition of the mixture and the size of the hemp shives fraction did not significantly influence energy consumption during densification. Energy values ranged from 1.234 × 10⁻^8^ J to 8.296 × 10⁻^8^ J. While the densification of pine after HWE was unsuccessful without ABS, preheating the mixtures with ABS facilitated the production of a uniform composite. The work required for densification ranged from 1.404 × 10⁻^5^ J to 2.711 × 10⁻^5^ J for fractions without ABS. For mixtures with ABS, the work required was 1.954 × 10⁻^5^ J for fraction 0 ÷ 0.4 (f1) and 0.042 × 10⁻^5^ J for fraction 0.4 ÷ 0.8.

## 1. Introduction

### Lignocellulosic Materials and Wood Plastic Composites—Use and Applications

Natural resources such as lignocellulosic materials have been with humanity since the beginning. In the past, it was primarily used as a fuel and a construction material. There has been a significant increase in its use over time. Modern technologies allow the refining and modifying of raw materials to be more widely used. Biodegradable plastics and biocomposites are gaining popularity as alternatives to traditional lignocellulosic materials, which are durable and resistant to biotic and abiotic factors [1]. The furniture industry has used wood-based panels for years, for example, particleboard, plywood, MDF, OSL, PSL, LVL, etc. [2,3]. Each of these types of boards is installed in most rooms, individually or in combination, to create solid connections. Wood particles are combined with resins at the proper pressure and temperature during the curing process to make these boards [4]. These materials are widely used in the furniture industry, where they have displaced all substitutes for a long time.

The pro-ecological policy may create problems with their future use in producing wood-based panels [5]. The European Union, like most countries, constantly tries to protect the environment by setting some restrictions on the acquisition of wood raw materials for the production of wood-based panels [6,7]. Changes in the furniture industry may occur quickly and unexpectedly, and substitutes for classic wood-based panels that are durable, practical, and affordable may be challenging to find. Materials engineering trends are increasingly moving towards green and sustainable solutions that minimize negative environmental impacts. Developing Wood Plastic Composites (WPC) is one of these innovative approaches. These materials combine wood with plastics and are gaining popularity for their mechanical and environmental properties. The advantages of WPCs over traditional building materials include their durability, low susceptibility to biological degradation, and recyclability. Compressive processes in biocomposites and the amount of mechanical energy consumed are important to WPC research. The constituent material moisture content, density, and ash content are essential physicochemical characteristics. Using plastics in the WPC improves its properties by providing durability and resistance to moisture and weathering [8,9]. The prepared research also examines how different factors, including biocomposite composition, influence the compaction process and final product strength.

Currently, there is growing interest in biocomposites combining polymers and lignocellulosic materials. Increasing numbers of companies are producing WPC (Wood Polymer Composites) products as evidence of this [10]. Composites became extremely popular during the 1960s. Innovative materials were developed with unusual properties that were difficult to achieve with traditional raw materials. Composites were expected to meet the demands of designers and constructors [11]. Combining lignocellulosic material and thermoplastics can produce composite materials with even better properties. During the processing, thermoplastics are manufactured, which can be used as a binder [12]. Thermoplastics are characterized by high strength and durability despite their origin from recovery. Thermoplastic polymers like ABS are highly mechanically strong and impact resistant, making them an ideal semi-finished product for hemp fibers.

Furthermore, because of their production process, shives have been widely recognized as waste; however, they are renewable [13,14,15]. These materials can be combined to create highly durable and lightweight products that are not only applicable to the furniture industry but also to other fields, such as automotive or construction. The versatile use of composites in the construction sector is due to their ability to withstand diverse climatic conditions [16].

Approximately six million hectares of land in Poland are planted with Scots pine (*Pinus sylvestris*), one of the most intensively exploited crops there [1]. Scots pine is ubiquitous in Polish and mixed forests, which explains its high occurrence rate. The potential to regenerate naturally and the economic value of deciduous forest trees create some financial support. The pine species have qualitative and strength properties, which make it a valued species [17]. The tree species determine the wood’s anatomical structure, which is directly related to its density. Among biomaterials, industrial hemp (*Cannabis sativa* L.) is also becoming increasingly popular [6,18]. In the past, it has spread to many parts of the world, including Poland. Hemp fibers have excellent strength properties and can be easily obtained from large-scale hemp cultivation plants. In Poland and worldwide, hemp is becoming increasingly popular as a potential building and furniture material [19,20]. Despite some legal restrictions, this material is becoming increasingly popular in the domestic market. As an alternative to traditional materials, hemp can also be used commercially, increasing the domestic supply of this raw material. Due to its numerous applications, Hemp is becoming increasingly popular as a lignocellulosic material. These composites are increasingly popular due to their durability and environmental friendliness, which hemp fibers possess.

The most common thermoplastics used to make WPC composites are polyethylene (PE), polypropylene (PP), or polyvinyl chloride (PVC). This study used ABS thermoplastic because of its recyclable properties and widespread availability. The plastic used in producing WPC plays a crucial role in shaping the final product, as it acts as a matrix that binds and stabilizes the lignin-cellulose materials. The thermoplastics used to make these composites are often recycled, which helps reduce plastic waste and promotes sustainability. Recycled plastics, such as PET bottles and industrial waste, can be used to produce WPC, which is both financially and environmentally beneficial. The acrylonitrile-butadiene-styrene copolymer, ABS, certainly deserves attention among the many polymers available. ABS is used in many polymer composites as a structural material and component. The popularity of ABS homopolymer can be attributed to its many favorable properties, such as high thermal and chemical durability, excellent dimensional stability, stiffness, and ease of processing. There is good reason to believe it has widespread use in various industries [21]. As an example of thermoplastics, or thermoplastic elastomers, ABS is one of them. The material can deform plastically under the influence of high temperatures. The material becomes soft during this process, allowing it to be molded and shaped as desired. Thermoplastics become solid, rigid bodies as soon as they cool down, enabling them to maintain their shape with great precision. These plastics have the advantage of being able to be plasticized repeatedly without affecting the material [22].

The wood plastic composites (WPC) are composite materials composed of lignocellulose and plastics. Combining these two components enables the production of materials that combine the attributes of wood and thermoplastics while eliminating many of their disadvantages, such as moisture absorption, deformation, and defects in the wood that affect its strength. Materials used in the manufacturing of WPC are not conventional. It is possible to produce composite materials using wood fibers, sawdust, shavings, and chips [23]. The filler can also be replaced with lignocellulosic materials, such as straw, plant stems, seed co-pops, and bamboo. Composites made of lignocellulosic material and plastic are becoming increasingly popular in various fields due to their unique qualities. WPC’s durability, lightweight, and weather resistance make it ideal for use in the construction industry. This material has many structural uses, including terraces, sidewalks, paths, stairs, floors, house facades, fences, and noise barriers. The aesthetic appearance of WPC makes them an attractive alternative to traditional materials [24]. Furniture manufacturers are increasingly using hemp in furniture production [19,20]. These fibers can be woven into composite boards characterized by light weight, strength, and flexibility. Especially ecological products-based furniture can also be aesthetic and functional.

Biocomposites can be manufactured with a blend of thermoplastics and hemp [25] or pine wood, according to studies published in the literature [26]. Composites based on hemp-ABS, for example, have been evaluated for their mechanical properties [8,27]. Our results show that hemp fibers can enhance ABS’s properties if processed correctly [28]. The durability of composites was also examined by environmental factors, including humidity and temperature [27]. Heat treatment is an effective method for improving the properties of pine wood [29], increasing its dimensional stability and moisture resistance. This research focused on preparing and characterizing a thermoplastic-polymer lignocellulosic biocomposite [23]. This research aims to contribute to the advancement of WPC technology by focusing on the preparation and characterization of a thermoplastic-polymer lignocellulosic biocomposite with varying proportions of pine chips (*Pinus sylvestris*) and hemp shives (*Cannabis sativa* L.) bound with an ABS regranulate. The study investigates the impact of ingredient proportions on the strength parameters of the resulting biocomposites. By conducting a thorough strength analysis and detailed statistical examination, this research provides a comprehensive understanding of their properties and application potential, ultimately promoting the development of sustainable and high-performance materials [23,30]. This in-depth analysis of ingredient parameters and proportions is crucial for optimizing composite technologies and materials.

The environmental friendliness of wood-plastic composites makes them more attractive to manufacturers looking for sustainable solutions. In the context of the goal above, evaluating mechanical energy consumption during the compaction process of these wood-plastic biocomposites with ABS thermoplastic is crucial for expanding their application areas and realizing their full potential. This investigation into energy efficiency aligns with the growing demand for sustainable manufacturing practices. WPCs offer inherent advantages due to their lightweight nature and ease of processing, potentially leading to reduced energy consumption compared to traditional materials [31]. The application can reduce weight in various structures, further enhancing energy efficiency. Therefore, this research on mechanical energy consumption in WPC production provides critical insights into the sustainability of this modern material, paving the way for its broader adoption in environmentally conscious industries. The prepared biocomposites will be rigorously analyzed regarding their strength properties and the parameters derived from this process, providing a comprehensive assessment of their performance and potential [31].

## 2. Material

### 2.1. Properties of Applied Materials

Materials are in high demand, requiring new solutions in the industry today [32]. Pine and hemp are considered lignocellulosic materials. The research aimed to prepare an environmentally friendly biocomposite that combines pine or hemp with thermoplastic. The materials used to make biocomposites were chips of pine or shives of hemp and the thermoplastic called acrylonitrile-butadiene-styrene (ABS) terpolymer. Despite being processed, regranulate maintains its properties since it is made from recycled plastics. The study used pine fractions divided into two groups with ranges of 0–1 and 1–4 mm. The binder used for this project was acrylonitrile-butadiene-styrene (ABS) terpolymer combined with shredded pine (*Pinus sylvestris* L.) wood. The materials were mixed by hand with a spatula. No specialized mixing or grounding equipment was used.

**Figure 1:**
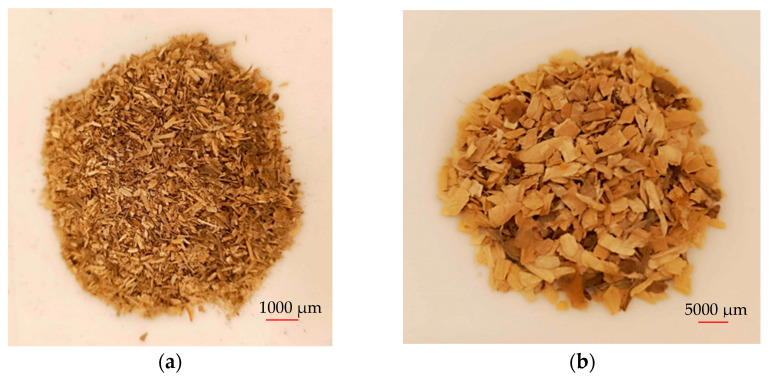

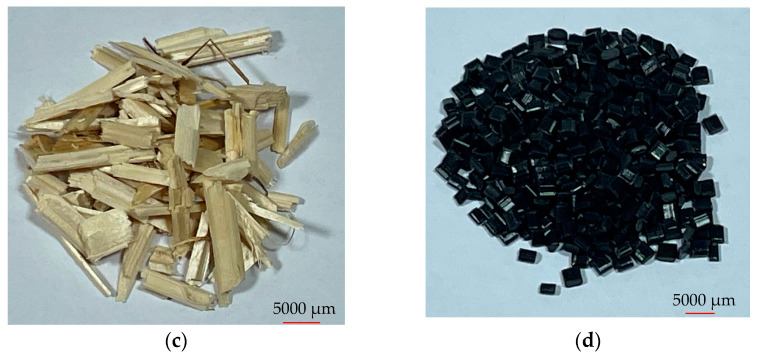
Material (**a**) Scots pine fraction *f1* (0 ÷ 1), (**b**) Scots pine fraction *f2* (1 ÷ 4), (**c**) hemp, (**d**) granulated ABS.

Additionally, the part of the material in mixed fraction form of hemp shives (*Cannabis sativa*) was also prepared by dividing it into two groups, each having a different fiber length (0–0.4 mm and 0.4–0.8 mm), and mixing them in a ratio of 50 to 50 and 25 to 75 percent with ABS regranulate. The use of regranulate is usually independent of its appearance. Small plastic cubes with a side dimension of 0.3 mm were used in this experiment. Materials characterization includes measurements of two key parameters, including a melt flow rate of 21 g of polymer per 10 min and an impact strength of 13 kJ⋅m^−2^. The materials obtained for the research are presented in Figure 1.

Scots pine wood chips are used as a building material because of their mass occurrence and characteristic properties. The versatility and wide range of applications of pine wood have given it a leading position in construction and furniture making. It is important to note that one of the advantages of pine wood is its ease of processing. The timber used has a relatively high degree of compressive strength, which creates a wide range of construction possibilities. A significant variation in density and strength characterizes the anisotropic structure of the material. The material has a considerable variation in thickness and strength because of its anisotropic structure. A limitation of using pine wood is its defects, such as knots, blue stains, and bark pockets. In the industrial aspect, an additional undesirable feature is hygroscopicity, manifested by a tendency to absorb moisture or give it away to the environment until it reaches a state of equilibrium between its moisture content and that of the environment [32].

Hemp is composed of fibers and the woody part of the stem created during the processing of hemp straw into fibers, called shives. Composite materials from hemp shives can be manufactured in various forms [10]. Shives, previously considered waste materials resulting from the processing of hemp straw, are now regarded as valuable commodities [11]. The hemp stalk comprises a wooden core and a fiber arranged along its stem that surrounds the wooden core. Shives are used to describe the pieces that are cut from the stem. The stem of the plant has a space in the middle. The use of hemp shives in combination with thermoplastic allows the creation of an eco-friendly thermoplastic with sought-after material properties.

Fractions of lignocellulosic material were combined with a thermoplastic polymer to create the composite material. The ability and capacity of thermoplastic to repeatedly convert from a solid state to a rubber-like state under heat is its most sought-after processing characteristic. The change in temperature affects the plasticity of the material used. Temperatures between 190 °C and 230 °C were considered most appropriate for this process. The acrylonitrile-butadiene-styrene terpolymer ABS can be produced by polymerizing 1,3-butadiene and copolymerizing acrylonitrile with styrene, followed by grafting the polybutadiene copolymer onto the acrylonitrile copolymer [12]. The combination of thermoplastic and lignocellulosic materials results in a material with valuable physical properties. In addition to improving the material’s moisture resistance, ABS increases its resistance to wood decomposition and facilitates processing. The strength and flexibility of the semi-finished product make it a good fit for applications that require strength and flexibility. Combining hemp and ABS thermoplastic can create a sustainable solution that meets both technical and ecological requirements and reduces the consumption of natural resources. Reducing non-renewable resources improves the mechanical properties and sustainability of the material. The sought-after mechanical properties are leading to the commercialization of WPC composites.

### 2.2. Samples Preparation

#### 2.2.1. The Fractions Separation

**Figure 2:**
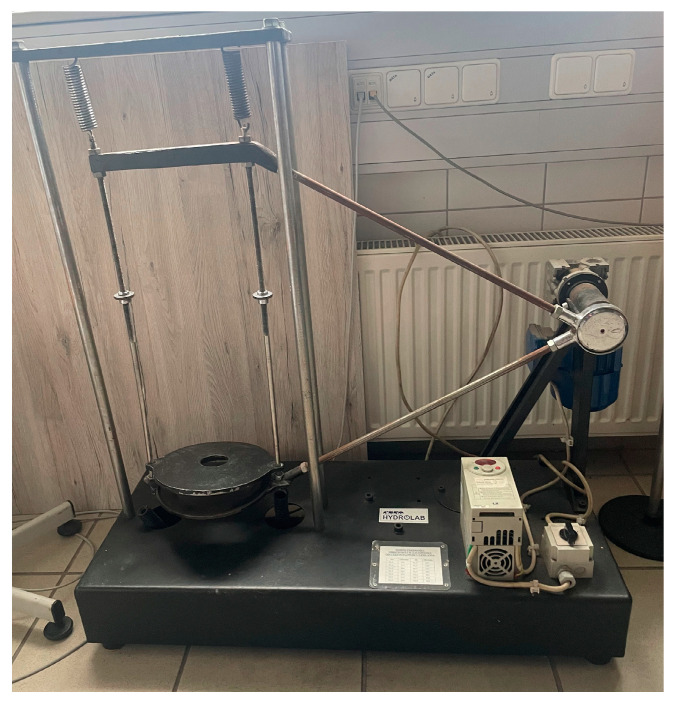
The machine that performs the screening process along with the established screens.

Research activities began with the preparation of intermediate products in a restful atmosphere. Steps were taken to pretreat pine wood and hemp shive. Shredding of the lignocellulosic material intended for compaction was a prelude to the controlled process. It was carried out in the laboratory using a shredder. The shredded lignocellulosic materials were sorted into fraction sizes before being prepared into a biocomposite. An orbital shaker from CBKO Hydrolab (Warsaw, Poland) was used to achieve the desired effect. Guided by ISO 17827-2:2016, the sieve separator divided the material into fractions according to the Polish standard. To achieve this, C.B. KO Hydrolab’s rotary shakers were employed, which enabled precise separation of the material in metal containers equipped with sieves of various mesh sizes [33]. The diameter of the shredded material, in each case, was necessary due to the technical possibilities of the test. The material was prepared to design a specially prepared compaction head with a piston. The machine that performs the screening process along with the established screens is presented in Figure 2.

**Figure 3:**
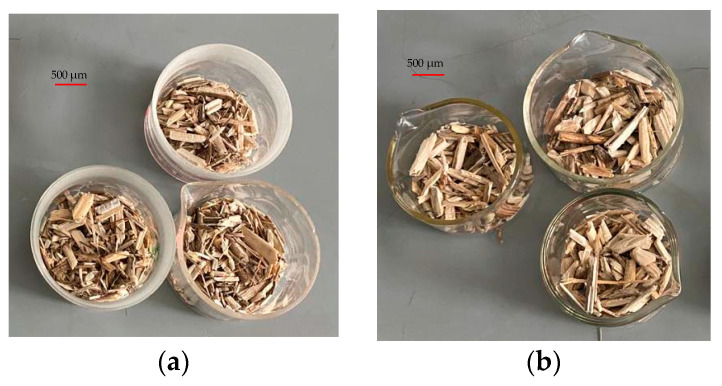
The fraction separates the test materials: (**a**) 0–0.4 and (**b**) 0.4–0.8.

Rotary shredders minimize impurities and heterogeneity to ensure high purity and size-specific parts. High-quality material was processed into biocomposite with the desired properties to meet the requirements of particular applications. Data from the literature [34] became the basis for establishing a range of fraction lengths from 0 to 4 mm, most suitable for producing a biocomposite with the expected shape and size. A fraction divided into two groups for Scots pine (0–1 and 1–4) and two groups for hemp fraction (0–0.4 and 0.4–0.8) was used for the study. The material was mixed based on its mass ratio after particle separation. The hemp shave materials separated by fraction are presented in Figure 3.

According to the purpose of the work, ABS mixtures with shredded particles of lignocellulosic materials were prepared as a base for further studies. It was unnecessary to sort the supplied ABS regranulate since it was in granule form. Regranulate ABS, in the form of a semi-finished product, has a fraction size of 0.3 mm. A mixture of this material was intended to be made with lignocellulosic biomass. To produce biocomposites with consistent properties, the parameters of each batch of material must adhere to standards during sorting, which is imperative for subsequent stages of production and testing. A biocomposite size of up to 14 mm in diameter was adopted. For further stages of biocomposite production, mixtures were prepared for both fractions of lignocellulosic raw material. A thorough sorting and preparation process ensures the precise measurement of quality and a high degree of consistency in the biocomposite, which is crucial for obtaining reproducible results and assessing the mechanical and thermal properties of the composite. To evaluate the mechanical and thermal properties of the obtained biocomposite, it is crucial to pay attention to the precise proportions of the components and homogeneity of the mixtures.

#### 2.2.2. Moisture Control

The samples were dried and weighed in precision-finished weighing vessels. Before the test, the weighing vessels (including the lids) had to be accurately weighed with an accuracy of 0.001 g. To minimize possible changes in moisture content, the prepared samples weighing about 5 g were placed in vessels under cover. In the next step, the ship was weighed again, with the samples, with an accuracy of 0.001 g. The samples in unique weighing vessels covered with lids were placed in the dryer after accurate weighing. Temperatures of 103 °C are used for drying. A set of instruments is required to use the dryer-weigher method, including a balance with a suitable measuring range, dryers, desiccators, and selected weighing cells. This apparatus allows accurate determination of moisture content. The samples can be dried using electric dryers, characterized by a temperature not below 120 °C and precision temperature control within two °C. Bimetal contact thermometers or mercury switches ensure restful thermoregulation conditions in the dryer. Once the dryer reaches a set temperature, it cuts the current flow and reopens. After that, the flow of current is cut. Temperatures are kept around set levels in this way. The dryers are equipped with vents. To ensure the dryer works correctly, they drain the water vapor and have a thermometer [27,35].

The specimens can be shaped differently, but too large dimensions can prolong drying. It is recommended to use rectangular samples of 2 × 2 × 3 cm to test wood’s mechanical properties and determine its moisture content. An appropriate sample size, measured by weight, should be selected to determine the moisture content of wood fragments such as sawdust, wood chips, or shavings. It is essential to weigh such a sample quickly and accurately because it has a large evaporation area. The drying process is monitored by performing regular check weighing. To minimize error during drying, weighing vessels should be covered with a lid before being removed from the dryer. The first check weighing is carried out about six hours after drying starts for softwood and about ten hours for hardwood. While weighing the vessel, it is necessary to cool it in a specialized device, in a desiccator, to a temperature of about 20 °C. After this process, the ship is again uncovered and placed in a unique drying device. The check weighing is recorded and repeated every two hours. The drying process is complete if the difference between the second from the end and the last weighing is not greater than 0.002 g. The disadvantage of this method is the need for an extended drying time for the samples. Five-gram samples should be dried for eight to sixteen hours till constant mass is achieved. The moisture content was determined, and the results were rounded to 0.1% using Formula (1):

(1) $${oneW}_{0}=\frac{G_{1}-G_{2}}{G_{2}-G}\cdot 100\%$$

where

*W_o_*—sample moisture, %,

*G*—weight of the weighing vessel, g,

*G*_1_—mass of the weighing vessel with the sample, before drying, g,

*G*_2_—weighing vessel with sample after drying to a constant weight, g.

The moisture content of the material needs to be monitored to ensure proper compaction conditions. The hygroscopicity parameter of the sample, which is determined by measuring its moisture content, was also assessed during the study. This test determines whether subsequent material processing should occur under appropriate conditions during the compaction process. Throughout the process, a constant moisture content must be maintained. As long as the material remains moist throughout the process, it should perform adequately. The literature states that the recommended moisture content for the raw material used in compacted biocomposites is 12–15% [34,36]. Samples of crushed mixtures from the literature with proposed moisture for natural materials (wood and hemp) were measured for moisture content during the study. The WPC products should contain about 2% storage moisture. To measure the moisture content of the samples, they were first dried in a laboratory chamber at 105 °C until they were scorched, then weighed as soon as they were removed. Using a laboratory cuvette, the samples were standardized for moisture content. It took a week for them to reach 12%. Densification does not require controlling the moisture level of the material before it is prepared. Scal-dryer Radwag WS30 (Radom, Poland) was used during the test.

#### 2.2.3. Specific Density Determination

**Figure 4:**
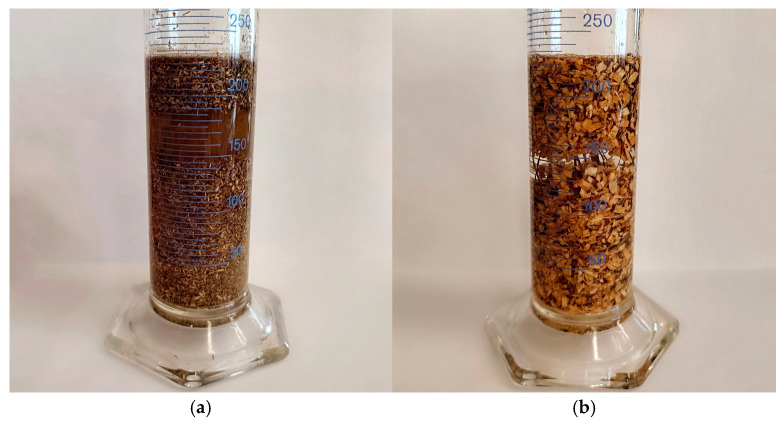
Specific density measuring station: (**a**) distilled water with pine fraction 0 ÷ 1 mm and (**b**) distilled water with pine fraction 1 ÷ 4 mm.

The study also aimed at determining specific density. The particular density and volumetric density of raw materials were measured during the characterization of the compaction process to obtain an overview of the studied raw materials. The methodology for calculating the specific density of biomass is based on the analysis of material compaction, considering the material’s internal porosity. The study assumes that the particular density of biomass is related to its ability to compact during the uniaxial compression process, the purpose of which is to reduce or eliminate intercellular gaps. Material density depends on how many gaps there are between the particles. The schematic of the specific density measuring set is presented in Figure 4.

The test is carried out by placing the test material in a vessel filled with distilled water. The material samples are immersed in distilled water, and the meniscus reading represents the specific volume level of the sample. A sign value is calculated based on the volumetric density of the material minus the area of the pores. The quotient of the mass of the measured raw material by volume, considering the external pores, corresponds to the specific density. According to the literature tests, water could not fill all intercellular spaces, resulting in a lower density value when measured with a helium pycnometer versus an aqueous solution. The entire measurement process is controllable and repeatable, resulting in accurate results. In addition, using the presented method and the density overcounting factor (χ) proposed in the literature [34,36] may allow a simpler, more efficient, and less expensive way to measure the specific density of materials.

### 2.3. Hot Water Extraction (HWE)

**Figure 5:**
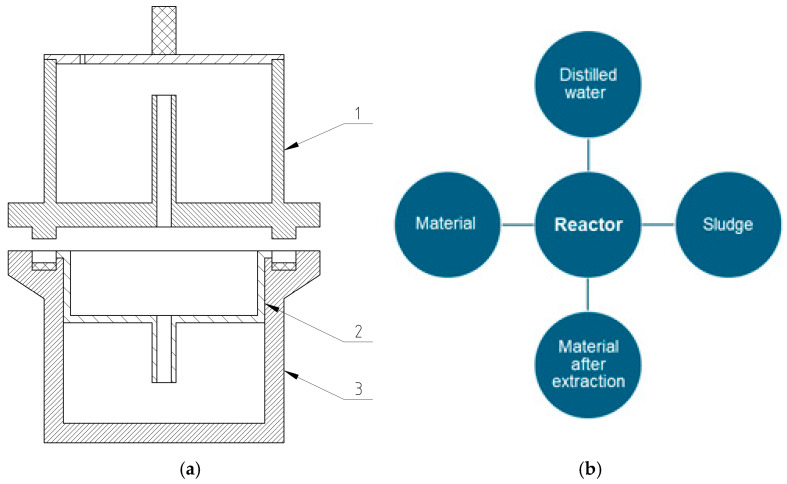
Hot water extraction: (**a**) set 1—container for extraction solution—reactor lid, 2 for chips, and 3 for reactor body, and (**b**) schematic of the HWE process layout.

Hot Water Extraction (HWE) was used to extract lignocellulosic material fractionated for the study. It has been determined that 10 g of the material has been weighed and placed in a container for testing the material before each process is initiated. The extraction was performed in a unique cylindrical device, using 400 g of distilled water under strictly controlled temperature (105 °C) and pressure (2 MPa). During the test of a single process, it took approximately 30 min for it to complete. The material and distilled water were replaced after the first extraction, and the process was repeated. After another exchange, three more repetitions of HWE were carried out on the following material. The hot water extraction set is shown in Figure 5.

According to the figure, the reactor lid is integrated into the solution container after extraction. Number 2 denotes the container for the chips, which undergo the HWE process. The reactor body was marked with the number 3. During the process, the container was refilled each time with distilled water, which, as a medium, flowed through the material during the process. Heat was being provided to the set at the bottom of the unit. High temperatures and pressures caused water to pass through the material, extracting the solution, which flowed into container 1. He was transferred to specially marked containers to dry the extracted material, which was then placed in a dryer. The post-extraction distilled water collected in the container was poured into labeled and weighed flasks. To obtain the weight of the sediment, an evaporation process was carried out on the flask containing the overflowing distilled water. The residue remaining after evaporation was brewed in a flask. The mass of the empty flask, weighed before extraction began, was subtracted from the result, obtaining the mass of the residue. In the same way as the native material, the post-extraction material was concentrated with ABS included. The intensity of the extraction process was controlled by repeated cycles of Hot Water Extraction (HWE) on the prepared raw material. The initial cycle of HWE I was conducted after filling the container with wood chips. After exchanging the analyzed material and distilled water, the HWE II process was repeated twice by adding only 400 mL of distilled water. Following the second cycle, the material and water were again replaced, and three more cycles of HWE III were conducted according to the methodology, supplementing the water by 400 mL for each time of change. This procedure allowed for a gradual increase in the extraction intensity, which allowed for more material recovery from the plant material with a higher degree of accuracy.

### 2.4. Structure and Function of the Compaction Head

**Figure 6:**
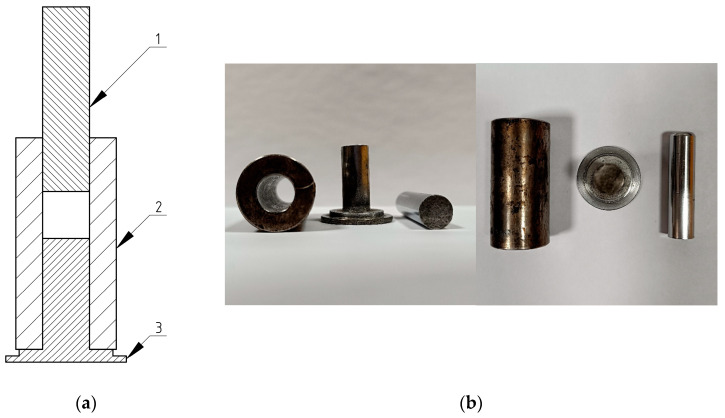
Diagram of the kit: (**a**) compaction set: 1-compaction head, 2-compaction sleeve, 3-support; (**b**) picture of compaction set.

The compaction set made from steel was designed to simulate the conditions compressing lignocellulosic material during actual compaction processes. The piston and chamber diameter were carefully selected to ensure that the unit pressure exerted on the material reached 3.5 MPa [36], a value known to be sufficient for binding the comminuted material together. The testing machine could generate a force of 100 kN, which provided the necessary compressive force to achieve this pressure unit. The integrated heating module played a vital role in the compaction process. By controlling the temperature of the material, it was possible to study the effects of heat on the compaction properties of lignocellulosic materials. The control panel could control the temperature precisely, ensuring the desired conditions were met throughout the testing process. The overall design of the test stand was carefully considered to ensure that the compaction process could be accurately monitored and controlled. The specialized equipment, including the compaction head, heating module, and control panel, provided the necessary tools for conducting comprehensive research into the compaction of lignocellulosic materials. The compaction set is illustrated in Figure 6.

Three structural elements form the prototype compaction head. The main one is a cylindrical sleeve in Figure 6, no. 2, mounted on a steel bracket in Figure 6, no. 3, and connecting the system to the testing machine via a steel shaft. The sleeve houses the movable component of the system, namely the compression head in Figure 6, no. 1. The compression heads are designed to achieve a certain unit pressure. The value of the maximum force generated by the compression machine (*F_max_*) and the size of the inner diameter of the compaction chamber had to be adjusted to achieve this value. Calculations were carried out to determine that the chamber’s inner diameter was equal to 11 mm. A cylinder with a height of 100 mm was included in the compaction set. The compaction of lignocellulosic materials at elevated temperatures is negatively affected by thermosensitizing factors. Compaction generates heat internally under stable conditions as well. The laboratory head is essential for maintaining the desired temperature in laboratories. A compaction head with an external heating unit was used to achieve laboratory conditions. Band heaters (GOGO-04349) (Warsaw, Poland) were used here. As a result, it was possible to determine the temperature to within one °C during the test. It ranged from 0 to 800 °C. The band radiator used here was 25 mm high. It was controlled by a thermocouple inside the heater through the 280-watt ESM-3710 controller. Digital readings of the controller gave an accuracy of 1%.

### 2.5. The Raw Material Compaction Process

**Figure 7:**
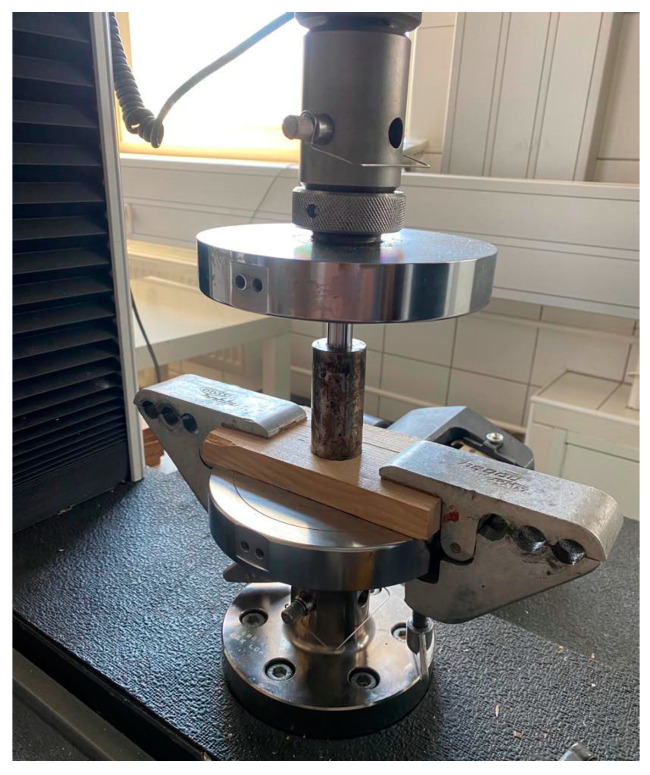
The particular prepared compacting head.

The laboratory process of preparing biocomposite materials requires precision and care. Shaking machines are used to separate hemp shives into fractions based on carefully selected screens. The next step is to thoroughly mix the two materials in appropriate proportions, which ensures even distribution of the components and, ultimately, the homogeneity of the composite. The thermoplastic ABS is heated to the appropriate plasticity in the next phase, facilitating the forming process. To ensure uniformity and density of the composite, the laboratory material is compacted in a testing machine using a specially designed compaction sleeve. Compression is followed by the sleeve’s tempering and cooling, stabilizing the material’s structure and properties. Technically advanced and precise, this process produces biocomposite properties that match expectations. Plasticized granulates play an important bonding role by replacing traditional adhesives, affecting the final product’s strength and durability. The present study implemented a model of a compaction head and connected it to a stress-testing machine. Compaction processes were carried out in the laboratory using a compaction head. Material parameters such as moisture content, granulometric composition, and process temperature had to be considered during the process. The compaction head was heated first in the test to ensure consistent temperature conditions throughout the experiment. The heating step was essential to simulate natural conditions accurately and achieve reliable results. The specially prepared compacting head is presented in Figure 7.

The research carried out was combined with the implementation of a model of a compaction head that cooperated with a stress testing machine. Controlled compaction processes in the laboratory were carried out with the help of the compaction above head. The process had several stages. The parameters of the material (moisture level and granulometric composition) and the method (temperature) were considered. The first stage introduced crushed material of a specific fraction and a specific percentage of ABS into the thickening sleeves. In the second phase, the compaction head and material were heated to a particular temperature in a dryer. In the third stage of the compaction process, the piston of the compaction head was locked in the upper part of the testing machine. The raw material was gradually compacted under pressure. After compaction, the piston was moved back to its starting position to remove the finished product. The product was removed using a compression head piston or an additional tool due to the product itself. The test rig explicitly designed for the study enabled measurements and control of the compaction parameters. The tests were carried out using a compaction head, the parameters of which did not differ significantly from those found in professional machines. Based on this, it can be concluded that the compaction head performs well in typical conditions where lignocellulosic materials are compacted. The final product had a diameter of about 14 mm.

### 2.6. Measuring the Ash Content

Inorganic components and purity of the material can be determined by the ash content, which is the residue after burning the material at high temperatures. The ash content of raw material is essential in determining its chemical composition. Identifying the ash content is a crucial component of quality control since it allows us to select materials that will produce composites with the desired properties. Determination of the ash content of the material involves ashing the sample at a high temperature and then accurately weighing the residue. This process is carried out in STOL’s muffle furnace, where the sample is subjected to a temperature of 800 °C. The material is wholly burned at this temperature, and the residue represents ash, which is then weighed on a precision analytical balance. The weighted ash represents the ash content of the sample being analyzed, which allows the amount of material to be determined relative to the weight of the entire sample. This method is a standard procedure used in chemical and physical analyses, po-posing to accurately determine the ash content of the material under study. Each sample was processed in a muffled oven for six hours. After charring, the samples were removed from the device, cooled, and accurately weighed. After washing, the samples were cooled in a desiccator, and the weight of the crucible, including the ash, was determined. A crucial part of this process is determining the ash content of the raw material, which is essential in assessing the material’s quantitative chemical composition. The remaining mass is converted to a percentage based on the results of the analysis. During the combustion test of the material, factors such as the duration of the process and temperature are essential. The listed parameters must be controlled to ensure uniform and reproducible incineration conditions. Despite similar chemical compositions, different material fractions may exhibit differences in material properties due to differences in consistency or density, which can also affect the combustion process and the final composition of the ash [37,38].

### 2.7. Statistical Analysis

The research was conducted to understand how the research materials were processed, which resulted in collecting a large amount of relevant data regarding the process by which the materials were processed. ANOVA (analysis of variance) has been used to characterize the correlation between the results obtained to support the primary statistical analysis. Analysis of variance (ANOVA) is a parametric statistical method for comparing more than two groups of data, separated based on one or more variables (univariate or multivariate). The primary purpose of variation analysis is to compare the dispersion (variance) of the dependent variable between groups that have been separated according to the independent variables. Scientists commonly compare averages between groups to determine the significance of differences [39,40].

During statistical analyses, the concept of variance is a fundamental measure of the variability of observed results. It can be interpreted as the arithmetic mean of the squares of the deviations of specific trait values versus the collective arithmetic mean [23,24]. It is worth noting that the value of the variance is always non-negative. The greater the value of the variance, the greater the variation that can be observed in the collective in the context of a given trait [39]. The study evaluated the effects of physical parameters on lignocellulosic biocomposites and thermoplastic ABS. As part of the method, Duncan’s test was used to divide the measured parameters into homogeneous groups [36]. The statistical analysis assumed that the other factors would remain constant. To obtain reliable conclusions from the experiment, excluding the influence of different variables is essential.

## 3. Results

### 3.1. Material Granulometric Properties

The granulometric properties of pine material play a key role in its use. The different fractions of lignocellulosic material can have different durability and processability, so defining the relevant fractions in the research process was essential. The first group of pine fractions used in the laboratory study was characterized by particle sizes falling within the range (0 ÷ 1 mm), while the second group included particles of size (1 ÷ 4 mm). An additional post-split group was introduced in the mass of hemp shives, where it was divided into groups of (0 ÷ 0.4 mm) and (0 ÷ 0.8 mm). Within the framework of the laboratory tests carried out, varied fractions of lignocellulosic material were used along with a binder in the form of ABS plastic with fractions ranging from 0 to 4 mm. ABS plastic is a thermoplastic material characterized by good mechanical strength, hardness, and chemical resistance. In addition, ABS is characterized by ease of molding, which is essential for processing this material. The samples were thoroughly mixed by hand before measurement to ensure homogeneity.

Several factors contributed to the segmentation of hemp shives into fraction groups, such as their mechanical properties and their influence on the parameters of the final biocomposite [34]. Smaller particles in the fraction can lead to a more homogeneous composite structure, while larger particles can add favorable mechanical properties, such as structural strength [36]. These two fractions made studying particle size effects on biocomposites possible. Particle size diversity will likely significantly affect biocomposite mechanical properties and stability. As a result of fractionation, the process of compaction of the raw material can be controlled more effectively, leading to improved consistency and homogeneity in the resulting biocomposites. The approach also allows for optimizing composite properties for specific applications, ensuring tailored performance characteristics. Advanced biocomposites with enhanced functionality and environmental benefits can be developed based on these findings. Research conducted on prepared material based on biocomposite production included an analysis of its moisture content as one critical step. According to current standards [33], precise measurements revealed that the tested material had a lower moisture content. It was necessary to increase the moisture content of the raw material to 12% after this value proved suboptimal. This procedure was carried out in laboratory cuvettes explicitly designed for this purpose, allowing precise humidity control. Increasing the moisture content of raw material enabled further processing, which improved the mechanical and dimensional properties of the biocomposites. Controlling moisture at this stage is extremely important, especially with compaction processes requiring adequate moisture to produce optimal results [41,42]. Adding 12% moisture to the shavings enhances the bond between the hemp fibers and the polymer matrix, increasing the final product’s strength. To determine how this factor influences the mechanical properties of biocomposites, we will divide the sample into fractions of different sizes. The physical parameters and moisture content of raw materials can be altered to improve the quality and properties of a biocomposite. To improve biocomposites’ mechanical and physical properties, further research will focus on the characterization and analysis of compaction processes.

The part of natural raw lignocellulosic materials is characterized by low weight and high mechanical strength. The hemp plant is an extremely promising intermediate for making innovative composite materials from renewable raw materials. The unique mechanical properties of these materials enable them to find applications in various fields. It is possible to create biocomposites from hemp by using a densification method that fully utilizes its beneficial properties. Hamp have a wide range of potential applications due to their unique properties. To increase the properties of the pine fraction, which lacks such high properties compared to hemp fibers, it was interesting to modify the material by using the HWE method before biocomposite production. The amount of ABS in the final product also adversely affected hemp biocomposites. According to the planned research, two mixtures were prepared, differing in the composition of material fractions. The first mixture was prepared with 50% ABS and 50% pine for fractions 0 ÷ 1 mm, interfering with fractions using the HWE method. The second mixture contained 50% ABS and 50% pine material 1 ÷ 4 mm, where the HWE method was also used for different cycles. These mixtures were subjected to a compaction process to achieve optimal density. In addition, hemp fractions of 0 ÷ 0.4 and 0.4 ÷ 0.8 mm were subjected to a compaction process in addition to 25 and 50% ABS. The goal was to analyze the effects of the combination of particle size on the compaction process of bio-composites. This method made it possible to thoroughly study the behavior of each fraction during the compaction process

### 3.2. The Specific Density Analysis

The research on the specific density of biomass focused on analyzing the material’s ability to compact by considering the material’s internal porosity. Biomass density was assumed to be based on the capacity of the material to compact during uniaxial compression to reduce or eliminate intercellular gaps. The filling of intercellular gaps defines the specific density of the raw material. Laboratory studies are essential in producing biocomposites and creating vegetation-based materials, which contribute to the more efficient use of the compaction process. During the experiment, biomass samples were immersed in distilled water as a medium, and the meniscus reading was the specific volume level of a given raw material. The quotient of the mass of the measured raw material by the volume, considering the external pores, corresponded to the specific density. The tests included samples of selected fractions for pine and hemp species. The particular density analysis of the compaction material involved a mathematical simulation of the packing of the material in the form of a biocomposite during compression. The packing factor, which is the product of the bulk density of the material, was used to evaluate the effectiveness of compaction *Ψ* [34,36]. This factor reflects the material’s ability to fill the available space under pressure. Multiple measurements of the volume of lignocellulosic material using a helium pycnometer allowed its packing factor to be determined at 0.62. This level’s specific density conversion factor was set for lignocellulosic materials with a fraction range of 0 ÷ 8 mm [34]. The device’s precision enabled accurate volume measurements, considering even the smallest pores in the plant material. The results provided valuable information about its structure and properties, which allowed for optimal adjustment of the packing factor to the specifics of the process. A high packing factor indicates the material tendency to arrange itself densely, which is beneficial in many applications, such as the production of biocomposites.

#### 3.2.1. Pine Specific Density

Analyzing the density of comminuted wood material is crucial in biocomposite production since it impacts the product’s mechanical and technological properties. Specific density measures how tightly packed particles are per volume and reflects the mass of the material. Higher density indicates greater material compactness and potentially better mechanical properties, such as strength and stiffness. This study analyzes the specific density of two Scots pine fractions, 0 ÷ 1 mm and 1 ÷ 4 mm, subjected to different stages of hydrodynamic water extraction (HWE). The research aims to determine the influence of particle size and pretreatment method on the specific density of the material, which will allow for optimizing the biocomposite production process with desired properties. The compaction process can be optimized by understanding the particular density characteristics of these fractions. The ability to customize biocomposites for specific applications and ensure their suitability for various applications requires this knowledge. The results of the comparison of shredded density for pine are presented in Table 1.

**Figure 8:**
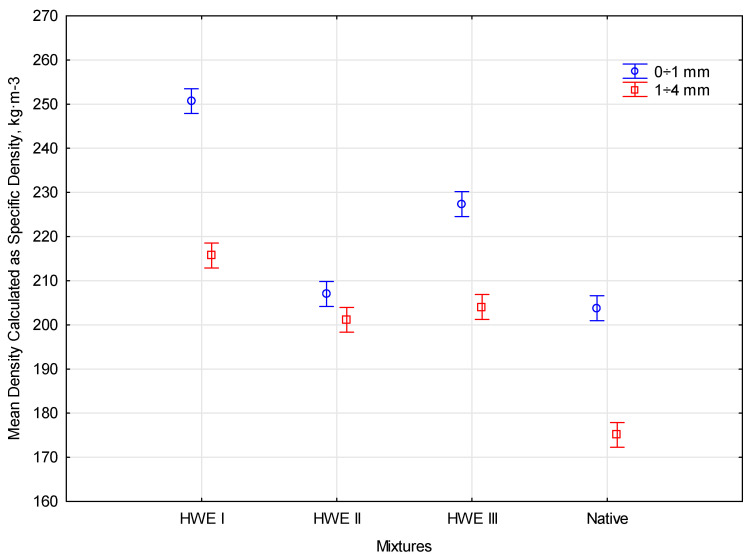
The average marginal relationships between the values calculated with the conversion coefficient (χ) depending on the size of the fractions.

Based on the results of the tests of pine fractions in the range of 0–1 and 1–4 for different effects of HWE, it was determined that the tested material had very similar properties. There were various mixtures for the native form and HWE I, HWE II, and HWE III treatments. The modification of HWE with an index indicated different stages of hydrodynamic water extraction. In the measurement process, it was noted that water did not penetrate the spaces between the individual materials. After adding the test procedure, a conversion factor (χ) was used to calculate the average density. This factor considered both internal and external pores filled with water, which was important for the accuracy of the measurements. The analysis revealed that fractions with smaller cross-sections fitted better together than those with larger cross-sections. This observation showed that smaller particles tended to form shorter distances between them. The analysis indicates that pine fractions 0 ÷ 1 and 1 ÷ 4 had similar physical properties. The specific density and ze value were also similar in both cases. There is a similarity between the structures and properties of the two pine fractions. The expected marginal average relationships between the values calculated with the conversion coefficient (χ) depending on the size of the fraction are presented in Figure 8.

The average marginal correlations between the values calculated with the conversion coefficient (*χ*) as a function of fraction size and HWE pretreatment were subjected to further statistical analysis. The measured parameters showed a correlation in the statistical analysis. The correlation between the studied parameters was confirmed by determining the statistical significance value below *p* = 0.5 ⋅ 10^−5^. The empirical value in this case was *F*(3, 16) = 44.184. As a result of the Duncan test being conducted, it was confirmed that there were significant differences between the measured parameters, causing them to be divided into two homogeneous groups.

#### 3.2.2. Hemp Specific Density

The compression of materials under pressure provides valuable insight into their behavior, which is an essential aspect of the development of biocomposites. This study prepared four distinct mixtures, each with varying proportions of hemp shave fractions and ABS thermoplastic (25% and 50% shares). The compaction behavior of these mixtures and the properties of the resulting materials were evaluated through compression testing. The analysis revealed significant differences in the compaction process and the final characteristics of the mixtures, highlighting the influence of particle size and ABS content. Compression effectiveness and properties of the materials varied considerably depending on the mixture composition. To achieve desired performance outcomes from biocomposite production, it is critical that components are selected and proportioned carefully. The relationship between mixture composition, compaction behavior, and material properties will be further explored. The results of the comparison of shredded density for pine are presented in Table 2.

**Figure 9:**
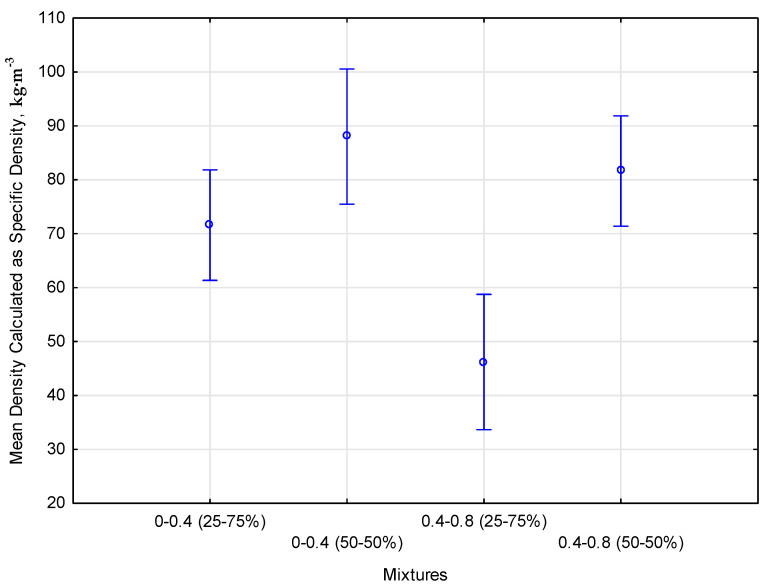
Statistical relationship between mean density calculated as specific density and type of mixture.

The compression tests of four mixtures, differentiated in terms of the composition of fractions 0 ÷ 0.4 and 0.4 ÷ 0.8 with ABS thermoplastic at 25% and 50% share, revealed a significant differentiation both in terms of the effectiveness of the compression process itself and the physical properties of the obtained materials. These results indicate significant differences in the behavior of materials depending on the ABS proportion, which may be important for their application in engineering and industrial practice. The total compression work, which measures the energy needed to compact the material, ranged from 1.234 × 10⁻^8^ J to 8.296 × 10⁻^8^ J, with the highest values recorded for mixtures with 50% ABS. The bulk density, indicating the degree of particle packing per unit volume before compression, was varied and ranged from 81.10 kg⋅m^−3^ for fraction 0.4 ÷ 0.8 with 25% ABS to 150.78 kg⋅m^−3^ for fraction 0 ÷ 0.4 with 50% ABS. As a result of the compression process, the density for all mixtures increased, maintaining a similar level of variability. The average density for the samples before compression was 119.75 kg⋅m^−3^. After compression, value increased to 132.89 kg⋅m^−3^, the largest increase in density was observed for the 0.4 ÷ 0.8 mixture with 50% ABS share. The specific density, taking into account both the solid material and internal and external pores, was lower than the bulk density, which indicates the porosity of the tested materials. The statistical relationship between the mean density calculated as specific density and the type of mixture is shown in Figure 9.

The research results were subjected to a one-way analysis of variance (ANOVA) to confirm the statistical relationship and post-hoc test. According to the methodology, the influence of the percentage share of ABS on the compaction process of biocomposite mixtures was assessed. The analysis compared the average values between groups and evaluated the statistical significance of the observed differences. In most cases, the results of the ANOVA analysis showed no statistically significant differences between the groups. This means that the differences in energy expenditure between the different mixtures were not large enough to be considered statistically significant. A detailed comparison of the average energy expenditure values between individual groups required the use of Duncan’s post-hoc test. This test allows for the identification of homogeneous groups, i.e., those that do not differ from each other in a statistically significant way. In one case, a difference was determined for the 0.4 ÷ 0.8 fraction, ABS 25%, separating the mixture into an individual homogeneous group. This means that changes in the ABS content in the tested mixtures did not significantly impact the compaction process in terms of energy consumption, except for one mixture. The results obtained suggest that the proportions of ABS in the mixtures can be adjusted to other criteria without affecting the energy efficiency of the compaction process.

### 3.3. The Process of Compaction of Pine with the Addition of ABS

#### 3.3.1. Pine Biocomposite Compaction Process

**Figure 10:**
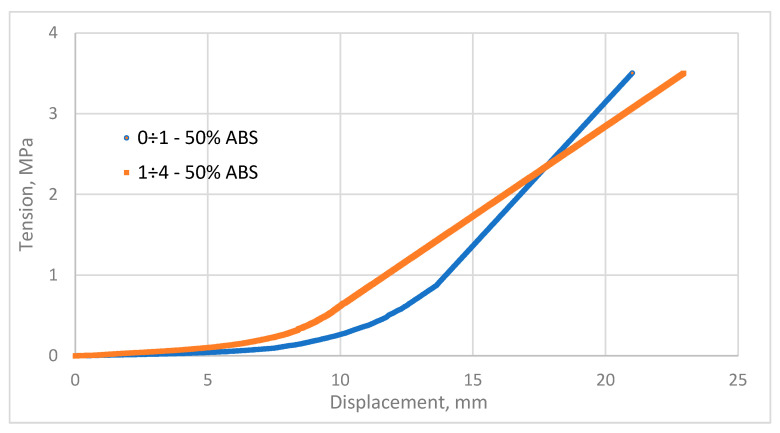
Characteristics of the biocomposite compaction process with 50% ABS.

The thickening of raw wood material with ABS thermoplastic was carried out in a specially prepared thickening setup equipped with a heating module and a control panel. The study showed that introducing a thermosensitizing agent and using appropriate laboratory equipment, such as a thickening head with an external heating unit, enabled the process temperature to be controlled with a 1 °C accuracy in the range from 0 to 800 °C. The prepared mixture, containing individual pine fractions 0 ÷ 1 and 1 ÷ 4 and 50% ABS, was subjected to thickening at a higher temperature (230 °C) to take advantage of the ter-mic properties of ABS. The thermal treatment was responsible for the plastic reaction of the ABS material. Separate fractions of pine 0–1 and 1–4, which were compacted with constant moisture content (12%), formed the base for this product. The pressure during the compaction process was about 3.5 MPa, corresponding to about 3.5 MPa, corresponding to the pressure generated during the compaction of particleboard. The compaction head, under laboratory conditions, was tested in accordance with the adopted parameters thanks to a properly designed test stand. The characteristics of the biocomposite compaction process with 50% ABS content for individual pine fractions are shown in Figure 10.

**Figure 11:**
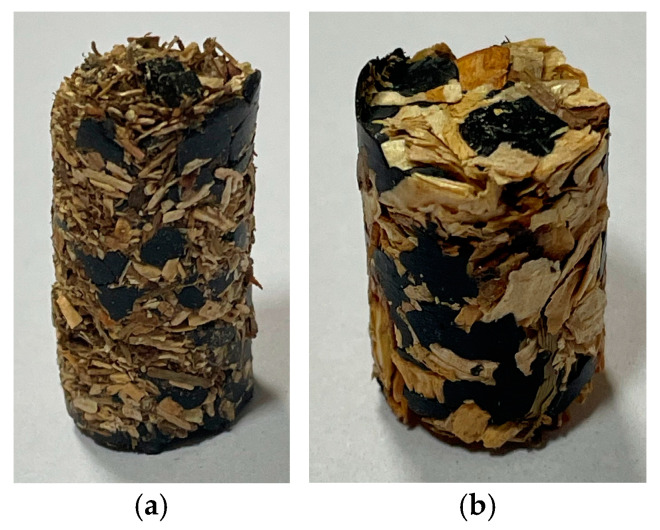
The mixtures after the compaction process (**a**) Scots pine of fraction 0 ÷ 1 with 50% ABS and (**b**) Scots pine of fraction 1 ÷ 4 with 50% ABS.

The graphs show that the large fraction is more susceptible to compaction than the smaller fraction. The head of the compaction device moves at an equal speed *v* = 5 mm⋅s^−1^. The pressure in both cases initially reached a close to zero value at the beginning of the experiment. In this process, two mixtures contain 50% ABS and individually 50% pine of fractions 0 ÷ 1 and 1 ÷ 4 and are mechanically compressed to remove the accumulated air that is pushed out. The minimum force required during this period was not determined by test equipment. The mixture had to undergo concrete compression, homogenization, and deformation to detect a noticeable compression force. The mixture of pine material of fraction 0 ÷ 1 with 50% ABS and the mixture of pine material of fraction 1 ÷ 4 with 50% ABS after the compaction process is shown in Figure 11.

The compaction process followed a similar trajectory for each case studied, as evidenced by the graph. Based on the pine fraction, the compaction force of the biocomposite with 50% ABS changes during the process. To make the thermoplastic material adhere better to the pine fractions, the mixture was heated before compaction. In compacting fractions 1 ÷ 4 with 50% ABS addition, the thermoplastic could not fill all the pores and spaces created. Different processes accompany the blending of different wood chips of different sizes. Comparing the graph of the course of compaction of fractions 0 ÷ 1 with 50% ABS addition and fractions 1 ÷ 4 with 50% ABS addition, it was found that the pressure during compaction of both mixtures increases mildly at the beginning. For fractions 0 ÷ 1, the pressure at the later stage of compaction increases sharply, while for 1 ÷ 4, it increases more gently.

#### 3.3.2. Hemp Biocomposite Compaction Process

**Figure 12:**
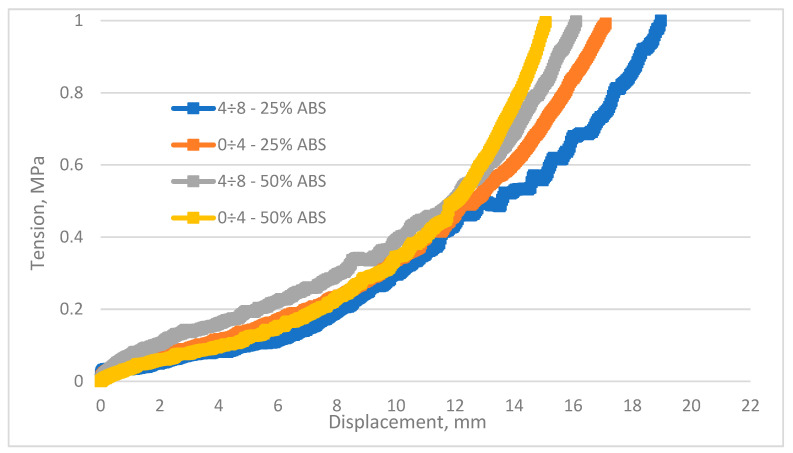
The characteristics of the compaction process of the biocomposite with 25 and 50% ABS for the different fractions of hempseed.

The biocomposite was manufactured by combining hemp shives with ABS regranulate to produce a product with the desired properties. In order to ensure the stability of the process and the quality of the final product, it was imperative to control the moisture level in the hemp shives. In a specially designed compacting attachment integrated with a heating module, hemp fractions 0 ÷ 0.4 mm and 0.4 ÷ 0.8 mm were thickened with a share of 25% or 50% of ABS additive. Using such a test rig allowed precise control of the process parameters and evaluation of the effectiveness of the compaction method. The thermoplastic was better bonded to the material fractions when the mixture was heated at 230 °C before compaction. The head of the compaction device moved at an equal speed of *v* = 5 mm⋅s^−1^, which caused the pressure to drop close to zero at the beginning of the experiment. Mechanical compression and the removal of accumulated air were achieved. The characteristics of the compaction process of the biocomposite with 25 and 50% ABS for the different fractions of hempseed were presented in Figure 12.

**Figure 13:**
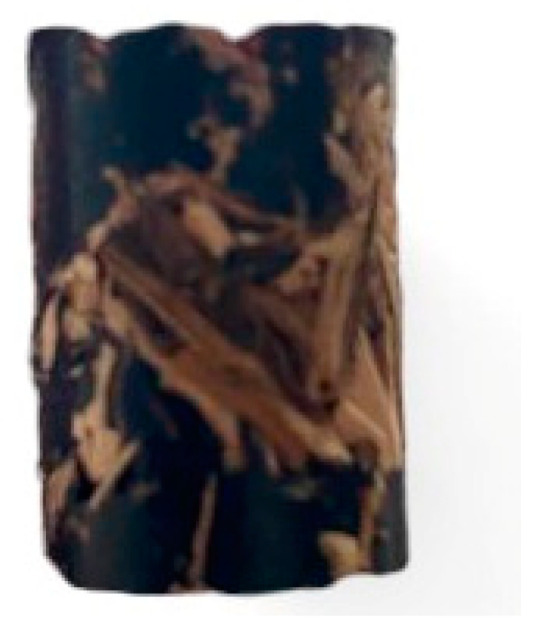
The product of the hemp compaction process with 50% ABS regranulate share and 0–4 mm of shives fraction.

Compared to samples with half ABS content, samples with 25% ABS content exhibit higher stresses. The higher stress values indicate that ABS content increases the force required to compact the material. In addition to the size of the material fraction, the material compaction process is also affected by the size of the fraction. The highest stress is generated at the end of compaction in materials with 50% ABS and 0–4 mm fraction. As a result, a product containing 50% thermoplastic but with a larger fraction (4–8 mm) will generate more stress than a product containing 25% ABS with a fraction of 0–4 mm. Because the thermoplastic content in the biocomposite is higher, compacting it will be more difficult and require more force. The lowest stress is generated by a sample with 25% ABS and a fraction size of 4–8 mm due to its less thermoplastic content and larger fraction size. The graph shows that higher ABS content and smaller fraction size produce better material compaction. Biocomposite compaction is significantly influenced by ABS content in this case. The final strength of the produced material can be increased by increasing ABS content, and smaller material particles allow better dissolution of bands, resulting in greater densification of the composite. The product of the hemp compaction process with 50% ABS regranulate share and 0–4 mm of shive fraction was presented in Figure 13.

Based on the data obtained from the four-measurement series, the piston displacement for the tested mixtures varied widely. Biocomposites were produced by compaction based on the mechanical properties of the resulting product. It is particularly important to note that the proportion of component shares affects stress values significantly. The series with a 50–50% ratio tend to have higher stress values than series with a 25–75% ratio. This course belongs to the series characterized by a small fraction range 0–0.4 mm that shows higher stress values than the other mixtures. After exceeding a certain displacement value of about 1.5 mm, a series of 0–0.4 mm with a 50–50% ratio shows a sharp increase in stress versus strain after exceeding a certain displacement value. The appearance of this phenomenon may indicate the material’s yield point, beyond which it undergoes permanent deformation.

### 3.4. Compression Process with Energy Characteristics

#### 3.4.1. Energy Expenditure in Biocomposite Compaction Process

Specific energy values were compiled for a mixture of ABS and raw material percentages to investigate the effect of fractions. Specific material parameters had to be characterized to calculate the total work value in the compaction process. The compaction of materials is usually described by a second-degree polynomial. The calculation of the range of the trend line is based on the least squares model. Based on this study, the coefficient of determination R^2^ did not exceed 0.99. The integral equation, a summary of the coefficients, and the overall value of compaction for specific parameters are shown in Table 3.

Compaction forces are best described statistically using a second-degree polynomial. The coefficient of determination *R*^2^ was about 0.99. The total work was calculated using 50% ABS in mixtures with materials and process parameters for the compaction process. The compaction factor can be calculated using distilled water and the density of the material before deformation to determine the amount of material compacted.

#### 3.4.2. Energy Expenditure in the Hemp Compaction Process

The research results provided valuable information on the behavior of ABS and hemp shive mixtures under stress, allowing for a better understanding of the biocomposite creation process. The analysis of the calculated energy values [36] for mixtures with 25% and 50% ABS content, divided by differences in hemp shive fraction size, allowed for the characterization of the effect of particle size on energy consumption during the process. The aim was to determine the work performed during the densification process by fitting trigonometric functions to the individual courses of each mixture. The calculations of the trend line range were based on the least squares method, and the coefficients of determination R^2^ confirmed the high quality of the fit, each time exceeding 0.95. In most cases, the densification of the mixtures was described by a second-degree polynomial, which allowed for a more accurate representation of the irregular pressure changes during the process. In the individual case, to describe the course of the mixture with the fraction 0–0.4 with 25% ABS, a sixth-degree polynomial was used, increasing the coefficient of determination R^2^ to 0.969. This allowed for the implementation of the assumption of maintaining the quality of the fit. According to the methodology, the obtained polynomial should be integrated to determine the energy value of the process. The integral equation, summary of coefficients, and the general value of densification for specific parameters, along with instructions for evaluating the coefficients, are presented in Table 4.

The total work was calculated in the densification process using 25 and 50% ABS in mixtures with fractional differentiation of hemp shives in the range from 0 to 0.4 mm and from 0.4 to 0.8 mm. The values of the total work performed during the densification process, depending on the displacement for different biocomposite mixtures, range from 1.234 × 10⁻^8^ J to 8.296 × 10⁻^8^ J. Differences in the energy requirements of the process can be observed depending on the material composition, which indicates the influence of the proportions and size of the fractions used in the mixtures. The analysis showed that using a second-degree polynomial to describe the densification forces was the right choice. The average coefficient of determination R^2^ was about 0.97, which indicates a very high accuracy of the fit of the theoretical model to the actual experimental data. The coefficient of determination R^2^, which assesses the fit of the theoretical model to the experimental data, oscillates between 0.9595 and 0.9846, confirming the fit’s high quality and the stability of the densification process. The estimated data are important for accurately assessing the efficiency of the densification process and for optimizing the biocomposite mixture in terms of its mechanical and operational properties. Thanks to these results, it is possible to precisely adjust the production parameters to the application needs of the composite.

## 4. Discussion

The research carried out concerned the preparation and characterization of selected physicochemical properties of lignocellulosic biocomposite with thermoplastic. In this study, ABS regranulate thermoplastic was used as a binder for the biocomposite at varying weight percentages of raw materials and binder. The fractions of lignocellulosic material were further divided into pine 0–1 and 1–4, and hemp 0–4 mm and 4–8 mm, and mixed in a 50–50% ratio with ABS regranulate, and hemp shivers in a 25–75% proportion. The compaction process of the biocomposites was statistically analyzed, as were the results of energy expenditure, which were statistically re-analyzed in detail. Statistical analysis allowed a comprehensive evaluation of their properties and potential practical applications. The analysis of the parameters and proportions of the components allows for the improvement of composite technologies and materials. The results obtained can be used to optimize the parameters of the biocomposite production process, which will translate into an improvement in their mechanical properties and increase the potential for their applications. Research was also conducted to create a new material that could be made from recycled or ecological materials [8,9]. We devised this design using wood and hemp shives in conjunction with ABS thermoplastic.

The hot water extraction (HWE) process alters the properties of wood, removing certain components and influencing its subsequent behavior. Adding thermoplastic polymers, such as acrylonitrile-lo-butadiene-styrene (ABS), can improve the mechanical properties of biocomposites. During the HWE process, cellulose is degraded due to high temperatures, distilled water, and pressure. The decrease in cellulose content may also be attributed to the selective extraction of other wood components, such as lignin and hemicelluloses, which are found alongside cellulose. As a binder substance, lignin helps provide stiffness and strength to wood cells. Densification of pine material after hot water extraction and manufacturing of composites using ABS and pine material provide new opportunities for utilizing wood waste in manufacturing and promote environmental protection and sustainable development. Biocomposites are produced with ABS regranulates, which reduce plastic waste as well as production costs, both of which are important economically [20]. Biocomposite properties may be affected by the presence of extractives in pine wood. The chemical composition of extractives can influence the adhesion of wood and thermoplastic additives. There is a need for further research on the effects of various extractive compounds on the performance and durability of pine-based biocomposites.

The energy consumption was measured by compacting pine mixtures of individual fractions with ABS at 230 °C. To achieve the right physical properties for the composite, moisture content had to be controlled. It was noted that the moisture content of the raw material plays an important role in the compaction process due to its influence on the plasticity of the material. Energy characterization was conducted to determine the energy consumption during compaction. The test material included blends of Scots pine (*Pinus sylvestris* L.) with 50% added thermoplastic called acrylonitrile-butadiene-styrene terpolymer (ABS) in proportions of 0 ÷ 1 with 50% ABS, 1 ÷ 4 with 50% ABS for the pine sample after hot water extraction (HWE). The results obtained after the compaction process showed that the amount of work done for each fraction without ABS addition ranged from 1.404 ⋅ 10^−5^ J to 2.711 ⋅ 10^−5^ J. For the mixture of 0 ÷ 1 with ABS, the value of work done was 1.954 ⋅ 10^−5^ J, and for the mixture of 1 ÷ 4 with ABS, the value of work done was 0.042 ⋅ 10^−5^ J. In literature, the range of values of the total work of pine wood compaction is from 65 J to 33 J in the moisture content range from 8% to 20%. For a moisture content of 12%, the work is 54 J [43].

The research results indicate a high potential for biocomposites made from hemp shives (*Cannabis sativa* L.) with ABS regranulate for use in, for example, the furniture and construction industries [23,44]. In accordance with research expectations, both the proportions of the ingredients and the length of the hemp shives fibers significantly affect the mechanical properties of the obtained material [19,45,46]. Composites with a higher share of shives (75%) and shorter fibers 0 ÷ 0.4 mm showed greater flexural strength and better elasticity compared to composites with a higher share of ABS (50%) and longer fibers (0.4 ÷ 0.8 mm), which confirms earlier literature reports on the influence of these parameters on the properties of lignocellulosic composites [36,45,47]. The porosity of anisotropic materials can be significant for the mechanical and thermal properties of the obtained materials and their potential applications. During the densification of the larger fraction of the material, the thermoplastic did not fill all the resulting pores and spaces, which indicates differences in the processes of joining fractions of different sizes. The research on biocomposites provided data on the behavior of specific mixtures and contributed to the development of a methodology for analyzing the densification processes of composite materials.

The development of research on biocomposites from lignocellulosic material with biodegradable thermoplastics should go beyond the optimization of the production process and focus on a holistic understanding of the impact of biocomposites on the environment throughout the entire product life cycle [8,19]. This means assessing the impact of acquiring raw materials through production and use to disposal or recycling. Developing advanced, efficient, and economically viable methods for recycling biocomposites is necessary to promote a closed-loop economy [48]. Discovering innovative applications for biocomposites, combined with intensified research on long-term durability in diverse environmental conditions, will ensure the reliability and safety of these materials [9]. The hemp shives and ABS regranulate are still promising raw materials, and continued research in this area will contribute to the further development of sustainable materials and technologies, supporting environmental protection [36,49]. The introduction of hemp shives as the main component of the biocomposite is in line with the research trend on the use of renewable and biodegradable raw materials in the production of composite materials [19,50,51]. Due to the material mechanical properties, such as high tensile strength and stiffness, hemp fibers are an attractive alternative to traditionally used fibers in the industry [19,51]. At the same time, the use of ABS regranulate, i.e., a material derived from recycling, fits into the idea of a closed-loop economy, which minimizes waste and maximizes the use of resources.

## 5. Conclusions

The study’s main purpose was to analyze the composition of the raw materials that underwent the compaction process, with particular attention to the possibility of producing plates from the obtained composites. The research also aimed to determine optimal process parameters and raw material preparation methods for biocomposite production. The conducted research indicates several important conclusions.

1. Studies involving composites made of lignocellulose and ABS thermoplastics showed significant effects on the densification of raw material composition, moisture content, and particle size. For example, the bulk density of hemp shives with 25% ABS increased from 81.10 kg⋅m^−3^ to 88.75 kg⋅m^−3^ after compression, while the bulk density of hemp shives with 50% ABS increased from 131.67 kg⋅m^−3^ to 153.02 kg⋅m^−3^ after compression.
2. The compaction behavior of pine material 0 ÷ 1 and 1 ÷ 4 or hemp material 0 ÷ 0.4 and 0.4 ÷ 0.8 was studied using ABS plastic. Heating ABS to 230 °C helps to make the form easy for the composite material to be plasticized. The process of compacting pine fractions with an ABS binder in a variety of proportions showed better physical properties for the finished product by plasticizing ABS.
3. Based on the results of the experiments, ABS did not contribute to a higher energy consumption during the compaction process. Increasing the moisture content of the raw material can improve plasticity and reduce the work required for compaction. The result is reduced work required for compaction, which leads to an efficient process and less energy consumption [43].
4. The porosity of hemp shives biocomposites plays an important role in shaping their properties. The average density of the samples increased from 119.75 kg⋅m^−3^ to 132.89 kg⋅m^−3^ after compression.
5. The largest increase in density was observed for the 0.4–0.8 mm mixture with 50% ABS share. These results highlight the need for further research on optimizing biocomposite components’ densification and mixing process to obtain more homogeneous and durable materials.
6. The research results provide practical information that can contribute to optimizing the biocomposite production process, increasing the potential for applications. For example, the total compression work for mixtures with 50% ABS ranged from 1.234 × 10^−8^ J to 8.296 × 10^−8^ J, and the average density of the samples after compression increased to 13,289 kg⋅m^−3^.
7. The research aligns with pro-ecological trends, offering sustainable alternatives to traditional materials and opening up new directions for developing innovative products. Further research is needed to optimize the composition of raw materials and the compaction process for improved mechanical and physical properties of biocomposites.

## Figures and Tables

**Table 1:** The results of the comparison of shredded density for pine.

Mixture	Mean Volumetric Density (SD) ^1^, kg·m^−3^	Mean Density After Compaction (SD) ^1^, kg·m^−3^	Mean Density Calculated as Specific Density (Internal and External Pores), kg·m^−3^
0 ÷ 1, Native	196.52 (4.75)	211.36 (26.42)	203.77 (2.38) ^bd^
0 ÷ 1, HWE I	180.38 (3.22)	194.37 (25.04)	250.69 (1.70) ^a^
0 ÷ 1, HWE II	149.50 (2.11)	165.75 (28.23)	206.97 (2.71) ^b^
0 ÷ 1, HWE III	162.48 (2.19)	180.44 (32.66)	227.37 (1.63) ^c^
1 ÷ 4, Native	160.38 (3.22)	177.38 (31.96)	175.06 (0.55) ^f^
1 ÷ 4, HWE I	157.57 (1.61)	177.01 (32.15)	215.70 (1.20) ^e^
1 ÷ 4, HWE II	148.09 (2.65)	170.02 (35.57)	201.14 (2.23) ^d^
1 ÷ 4, HWE III	146.34 (1.05)	165.87 (34.74)	204.05 (1.78) ^bd^

^1^ SD—standard deviation, ^a,b,c,d,e,f^—homogeneous groups.

**Table 2:** The results of the comparison of shredded density for hemp.

Mixture	Mean Volumetric Density (SD) ^1^, kg·m^−3^	Mean Density After Compaction (SD) ^1^, kg·m^−3^	Mean Density Calculated as Specific Density (Internal and External Pores) (SD) ^1^, kg·m^−3^
0 ÷ 0.4, ABS 25%	115.45 (2.15)	129.98 (2.16)	71.58 (1.34) ^a^
0 ÷ 0.4, ABS 50%	150.78 (17.20)	159.80 (17.11)	93.48 (10.67) ^a^
0.4 ÷ 0.8, ABS 25%	81.10 (12.13)	88.75 (12.10)	50.28 (7.52) ^b^
0.4 ÷ 0.8, ABS 50%	131.67 (17.99)	153.02 (16.24)	81.63 (11.15) ^a^

^1^ SD—standard deviation, ^a,b^—homogeneous groups.

**Table 3:** The integral equation, a summary of the coefficients, and the overall value of compaction for specific parameters.

Total Work Calculated Under Specified Conditions *W_(τ, φ)_*	Determination Coefficient *R*^2^ (SD)	Displacement l (SD), mm	Total Compaction Work, J (SD)
$W_{\left(f_{1},ABS\;50\%\right)}=\int_{0}^{0.001\cdot l}0.0122x^{2}-0.0887x$	0.992 (0.04)	21.01 (0.023)	1.95 × 10^−5^ (1.032 × 10^−5^) ^a^
$W_{\left(f_{2},ABS\;50\%\right)}=\int_{0}^{0.001\cdot l}0.0072x^{2}-0.0017x$	0.993 (0.06)	22.94 (0.427)	4.20 × 10^−7^ (0.633 × 10^−7^) ^b^

SD—standard deviation, ^a,b^—homogeneous groups.

**Table 4:** The calculations of total compaction work using the integral equation.

Total Work Calculated Under Specified Conditions *W_(τ, φ)_*	Determination Coefficient *R*^2^ (SD)	Displacement l (SD), mm	Total Compaction Work, J (SD)
$W_{\left(f_{1},ABS\;25\%\right)}=\int_{0}^{0.001\cdot l}-2\times 10^{-5}x^{6}+0.0004x^{5}-0.0028x^{4}+0.0088x^{3}-0.01x^{2}+0.0035x$	0.9690 (0.0335)	6.93 (0.001)	8.296 × 10^−8^ (7.043 × 10^−8^) ^a^
$W_{\left(f_{1},ABS\;50\%\right)}=\int_{0}^{0.001\cdot l}0.0139x^{2}-0.0123x$	0.9717 (0.0051)	3.50 (0.033)	7.514 × 10^−8^ (2.572 × 10^−7^) ^a^
$W_{\left(f_{2},ABS\;25\%\right)}=\int_{0}^{0.001\cdot l}7\times 10^{-5}x^{2}+0.0011x$	0.9595 (0.0028)	5.34 (0.009)	1.571 × 10^−8^ (4.735 × 10^−7^) ^a^
$W_{\left(f_{2},ABS\;50\%\right)}=\int_{0}^{0.001\cdot l}6\times {10^{-4}x}^{2}+0.0002x$	0.9846 (0.0461)	8.65 (1.299)	1.234 × 10^−8^ (6.399 × 10^−5^) ^a^

SD—standard deviation, ^a^—homogeneous groups.

## Data Availability

The original contributions presented in the study are included in the article, further inquiries can be directed to the corresponding author.
